# Reliability of hand held Doppler use in podiatrists

**DOI:** 10.1186/1757-1146-8-S2-O7

**Published:** 2015-09-22

**Authors:** Peta Craike, Bella Schlachter, Patrick Tehan, Kate Carroll, Krista Sturday, Vivienne Chuter

**Affiliations:** 1 Faculty of Health & Medicine, University of Newcastle, Ourimbah, NSW, 2258, Australia; 2 Port Stephens Podiatry, Nelson Bay, NSW, 2315, Australia; 3 Hunter New England Area Health Service, Podiatry & Footcare Department, Newcastle, NSW, 2300, Australia

**Keywords:** Vascular assessment, Hand-held Doppler, inter-tester reliability, intra-tester reliability

## Background

Hand held Doppler examination is a frequently used non-invasive vascular assessment utilised by podiatrists. Despite this, Doppler's reliability has not been thoroughly investigated. Given the importance of Doppler in completing a vascular assessment of the lower limb, it is essential to determine the reliability of the testing method in practicing podiatrists.

## Methods

This was a multi-centre inter-tester and intra-tester reliability study. Four podiatrists participated in this study, two public and two private practitioners. Three aspects of Doppler use were examined; the clinical Doppler technique, the evaluation of Doppler audio sounds and qualitative evaluation of Doppler waveforms. Participants meeting current guidelines for vascular screening attended two testing sessions, one week apart at either the private practice (N=32), or the public practice (N=31). Two podiatrists (either public or private) assessed their Doppler waveforms, and rated them as mono-phasic or multi-phasic. Podiatrists were also then required to analyse 30 waveform tracings chosen at random by the researchers, and 30 audio recordings of Doppler sounds recorded by the researchers. Cohen's Kappa (K) statistics were used to calculate inter and intra tester reliability using SPSS version 19.

## Results

Evaluation of waveform tracings demonstrated highest reliability, with inter-tester reliability ranging from K 0.77 to 0.90, and intra-tester reliability from K 0.81 to 1.00. The public podiatrists showed higher reliability in audio examination (inter-tester reliability K 0.61, intra-tester reliability K 1.00) compared to the private podiatrists (inter-tester reliability K 0.31, intra-tester reliability K 0.53). Clinical use of Doppler demonstrated the lowest reliability for both pairs of clinicians (inter-tester reliability K 0.20 to 0.24, and intra-tester reliability K 0.27 to 0.42).

## Conclusions

There is a need for ongoing education for podiatrists using Doppler in clinical practice, as the reliability of the clinical use of the Doppler was low. This indicates that technique could be an issue. There is also a need to evaluate if hand held Doppler equipment is suitable for use in the podiatry patient cohort.

